# Accurate and Robust Prediction of Genetic Relationship from Whole-Genome Sequences

**DOI:** 10.1371/journal.pone.0085437

**Published:** 2014-02-28

**Authors:** Hong Li, Gustavo Glusman, Chad Huff, Juan Caballero, Jared C. Roach

**Affiliations:** 1 Institute for Systems Biology, Seattle, Washington, United States of America; 2 Department of Epidemiology, University of Texas M. D. Anderson Cancer Center, Houston, Texas, United States of America; University of Southern California, United States of America

## Abstract

Computing the genetic relationship between two humans is important to studies in genetics, genomics, genealogy, and forensics. Relationship algorithms may be sensitive to noise, such as that arising from sequencing errors or imperfect reference genomes. We developed an algorithm for estimation of genetic relationship by averaged blocks (GRAB) that is designed for whole-genome sequencing (WGS) data. GRAB segments the genome into blocks, calculates the fraction of blocks sharing identity, and then uses a classification tree to infer 1st- to 5th- degree relationships and unrelated individuals. We evaluated GRAB on simulated and real sequenced families, and compared it with other software. GRAB achieves similar performance, and does not require knowledge of population background or phasing. GRAB can be used in workflows for identifying unreported relationships, validating reported relationships in family-based studies, and detection of sample-tracking errors or duplicate inclusion. The software is available at familygenomics.systemsbiology.net/grab.

## Introduction

Two individuals are related if they share a recent common ancestor. Knowledge of genetic relationships is crucial to genetic studies — including linkage analysis and heritability estimation. Related individuals needed to be removed in population-based association study to avoid bias [1], [2]. For example, software PRIMUS was developed to find the maximum set of unrelated individuals given a user-defined threshold of relatedness [3]. Genetic relationships derived from reported pedigree structures may be incorrect due to unknown relationships between founders, non-paternity, adoption or sample labeling errors [4], [5]. Therefore relationships need to be validated using genotype data.

Many existing methods estimate relationships from identical-by-state (IBS) or identical-by-descent (IBD) estimates between two individuals [6]. PLINK calculates IBS probability with a hidden Markov model [7]. KING provides a quick method for estimating a kinship coefficient which correlates with the degree of relationship [8]. SNPduo visualizes IBS patterns from meiotic crossover points in siblings to characterize relationships between individuals [9]. As relationship distances increase, variances increase in these metrics, so simple algorithms that rely on genome-wide estimates them are best suited for estimating close relationships. A more sophisticated approach, ERSA [10], employs a maximum-likelihood method to integrate more information from IBD segments reported from an input IBD algorithm. ERSA extends the range of relationship estimation to up to thirteenth degree relatives. The performance of ERSA relies on the accuracy of IBD algorithms. IBD algorithms improve with phased data, knowledge of genetic distances between variants (e.g., in centimorgans), and population allele frequencies.

Here we present GRAB, an algorithm for accurate estimation of genetic relationships by focusing on the distribution of IBS in segmented windows. GRAB works well with whole-genome sequencing (WGS) because it averages local noise across short blocks with little loss in measuring true signal, which is derived from correctly called ancestral variants aligned to an accurate reference genome. In essence, it works much like a “low pass” filter in electronics. Genome-wide metrics such as average IBS are not able to fully take advantage of this true signal. We tested GRAB on real and simulated families with various levels of sequencing error, and found it can accurate predict 1^st^-degree to 5^th^-degree relationship and robust to sequencing error.

## Methods

### Design

GRAB was designed both to address new aspects of WGS data as well as for simplicity and computational speed. WGS data tends to be more noisy than classical genotyping array data, in part because classical arrays were designed based on a carefully chosen set of positions that passed numerous rounds of quality control including demonstration of Hardy-Weinberg equilibrium in particular populations. GRAB addresses noise in WGS by averaging signal over blocks of positions. To enable averaging the genotype signal across sufficient markers, GRAB segments genomes into non-overlapping windows of 1 Mb (for WGS) or 2 Mb (for genotyping panels). These intervals were chosen empirically to provide a reasonable balance of sensitivity and specificity across a range of datasets. For WGS, more than 2700 windows were obtained that contained at least one single nucleotide variant (SNV). SNVs within a window are compared between pairs of individuals and classified into three groups: IBS2, when the genotypes of the two individuals are identical; IBS1, when they share exactly one allele; and IBS0, when there are no alleles in common. The fractions of SNVs in each IBS group are calculated for each window (called P0 for the IBS0 group, P1 for the IBS1 group, and P2 for the IBS2 group).

After computing P0, P1, and P2 for each window, GRAB classifies that window either as an identity window (IW) or not based on P0 (less than P0_cutoff_). P0_cutoff_ is chosen as a function of the parameter ‘sequencing error’ (SE). Sequencing error can be determined by replicate sequencing of identical sequences [11]; it is known precisely in simulated data. We explored values of P0_cutoff_ between 1-(1-SE)^4^ and 1-(1-SE)^2^. We set P0_cutoff_ to (0, 0.004, 0.01, 0.015) when SE is (0, 0.001, 0.005, 0.01) in simulated data.

**Figure 1:**
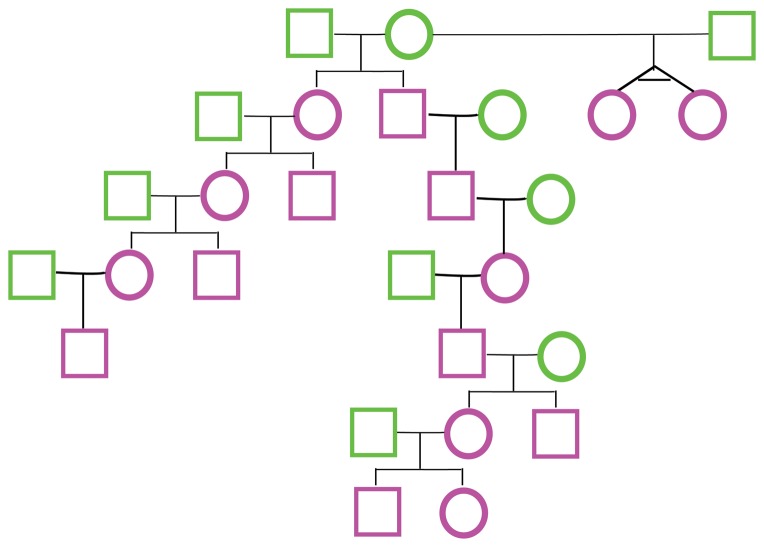
A simulated 26-member, 7-generation pedigree. Green symbols indicate founders that were sequenced by CGI, and purple ones indicate children whose genotyping were simulated. The topology of the pedigree was chosen to enable testing of diverse relationship estimations.

**Figure 2:**
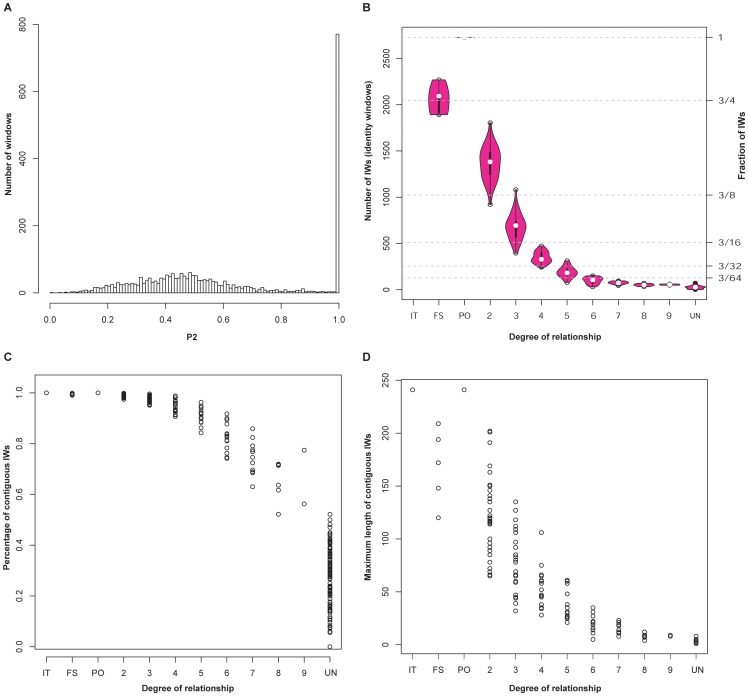
IBS percentage in different relationships of simulated families. For this visualization, the sequencing error (SE) parameter was set to zero. (A) Distribution of P2 in an example sibling pair. Siblings have much of the genome that is easily detectable as IBD2, which GRAB detects through a large number of windows with a very high P2 statistic. (B) Number of identity windows (IWs) between pairs of individuals, decreasing with increased relationship degree. (C) Percentage of contiguous IWs. A contiguous IW is any IW adjacent to another IW. Unrelated individuals have fewer contiguous IWs than relatives. (D) Maximum length of a set of contiguous IWs. This length tends to be shorter in distant genetic relationships than close relationships. IT: identical twin. FS: full sibling. PO: parent offspring. UN: unrelated individuals. UD: unknown distance.

One simulated family (Figure 1) was used to train the model and investigate the effect of sequencing error. Programs for simulating WGS families are available at (github.com/caballero/FakeFamily). The distribution of P2 is very different between pairs of siblings and other pairs of individuals. The sibling-pair distribution has a large component at high P2 (Figure 2A), indicating genomic segments that were inherited identically from both parents. We include the twin relationship or comparison of self with self in this group. For all other relationships such a large P2 component is not observed, GRAB extracts relationship information from the fraction of IWs. For parent-offspring pairs this fraction is approximately 1 (almost all genomic windows are IW), and it decreases with more distant relationships (Figure 2B). More distant relationships have fewer and less contiguous IWs (Figure 2C,D). The pattern of the number of IWs is similar for varying values of SE and P0_cutoff_, enabling use of a simple classification tree across a diverse range of parameters, including the number and length of contiguous IWs.

**Figure 3:**
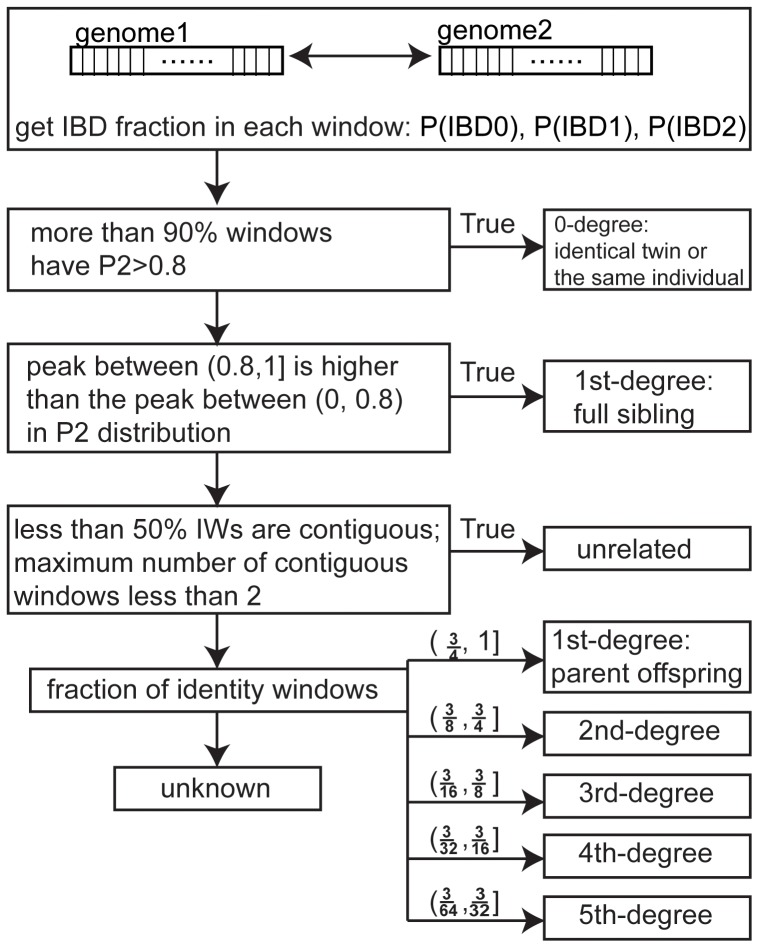
GRAB Workflow.

GRAB employs such a classification tree to estimate relationship (Figure 3). Degree of relationship is defined as the combined number of generations separating individuals from their ancestors [10]. Two metrics defined from the distribution of P2 are used to predict identical-twin and full-sibling relationships: the number of windows with P2 within (0.8, 1] and a logic value indicating whether the peak between (0.8, 1] is higher than the peak between (0, 0.8]. Three additional metrics used in the classification tree are the number of IWs, the percentage, and maximal length of contiguous IWs. They were used to estimate unrelated individuals and 1st-degree to 5th-degree relationship.

### Implementation

GRAB is stand-alone Perl software suitable for integration into high-throughput workflows. It accepts several input formats: PED+MAP or TPED [7], Genome Variation Format (GVF) [12], Variant Call Format (VCF) [13] and the ‘varfile’ format of Complete Genomics, Inc. (CGI). GRAB is available at familygenomics.systemsbiology.net/grab.

### Comparison with other algorithms

Whole-genome sequenced families were used as a test dataset. Input was filtered to include only full-called biallelic SNVs. PLINK [7] and KING [8] take PED+MAP file as input, and output the percentage of IBS (KING also estimate kingship coefficient). ERSA [10] takes IBD segments as input and uses a likelihood model to estimate relationship. Two IBD detection methods are used for ERSA: GERMLINE [14] and ISCA [15]. GERMLINE can be used on both unphased and phased data. It performs better on phased genotype than unphased data. However, phasing needs ancestor information, a large number of unrelated control individuals and longer running time.

## Results

### Software validation

To test the performance of GRAB we analyzed four datasets: 1) 182 individuals from 28 families that are whole-genome sequenced by CGI (∼3.5 million SNVs in each individual), 2) one family with 172 members genotyped by Affymetrix (Array version 6.0; ∼0.8 million nominal SNVs), 3) five WGS-simulated families with up to 15th-degree relationships, and 4) the same five simulated families restricted to Affymetrix array positions.

Most of the estimated genetic relationships by GRAB were consistent with the reported relationships in real families. Manual verification revealed that for all the discordant 1st-degree relationships, the GRAB predictions were confirmed to be correct: the discrepancies were caused by incorrect labeling of samples or reported pedigree structure. Such errors may be common when complex pedigrees are studied. Estimating genetic relationships is a very important quality control step in family genome studies. Before further measuring the performance of GRAB and other software, we corrected the reported pedigree structures to eliminate these discrepancies.

GRAB obtained exact predictions for 97% of 2nd-degree, 93% of 3rd-degree, and 97% of 4th-degree relationships in real WGS families (Table 1). For genotyping array data, GRAB's relationship accuracy is slightly lower than on WGS data; 67% of 5th-degree relationships were exactly predicted (Table 1). When not exact, the relationship was usually predicted to within one degree of the correct value. For example, 99% of 5th-degree relationships were predicted as fourth degree, fifth degree, or ‘related, more distance’. GRAB's relationship accuracy was better on real families than on simulated families (Table 2). All 1st-degree pairs were exactly predicted in simulated families, including distinctions between full-sibling and parent-offspring relationships. GRAB obtained 100% accuracy for unrelated individuals, 95% accuracy for 2nd-degree, and ∼60% for 5th-degree relationships (Table 2). Even when increasing the per-SNV error rate to 0.01, GRAB could achieve similar performance by adjusting parameter ‘P0_cutoff_’ accordingly.

**Table 1:** Fraction of correct predictions for real families.

Real families	Full sibling	Parent offspring	2nd-degree	3rd-degree	4th-degree	5th-degree	unrelated
WGS	1	1	0.97 (1)	0.93 (1)	0.97 (1)	0.57 (0.86)	0.98
Affy	1	0.91 (1)	0.875 (1)	0.89 (1)	0.85 (1)	0.67 (0.99)	

Values in parentheses are the percentage of predictions within one degree of the true relationship.

**Table 2:** Fraction of correct predictions for simulated families.

Error rate	P0_cutoff_	Marker	Full sibling	Parent offspring	2nd-degree	3rd-degree	4th-degree	5th-degree	unrelated
0	0	WGS	1	1	0.96 (1)	0.82 (1)	0.66 (1)	0.52 (1)	1
0	0	Affy	1	1	0.95 (1)	0.80 (1)	0.65 (1)	0.52 (0.99)	0.97
0.001	0.004	WGS	1	1	0.97 (1)	0.84 (1)	0.71 (0.99)	0.57 (1)	1
0.005	0.01	WGS	1	1	0.97 (1)	0.88 (1)	0.79 (0.99)	0.65 (0.97)	1
0.01	0.015	WGS	1	1	0.97 (1)	0.88 (1)	0.82 (1)	0.59 (0.95)	1

P0_cutoff_ is set based on per-SNV error rate. Values in parentheses are the percentage of predictions within one degree of the true relationship.

GRAB trades distant-relationship sensitivity for close-relationship accuracy. This trade is effectuated by employing a long segmentation block length that is robust to sequencing noise. GRAB successfully estimates up to 5th-degree relationships and classifies more distant relationships as unrelated (‘UN’) or unknown distance (‘UD’), which indicates a relationship without a prediction of degree). 94% of 6^th^-degree and 78% of 7^th^-degree relationships were predicted as related in simulated WGS families (sequencing error parameter: 0.001). In summary, GRAB performs exceptionally well in detecting and estimating close relationships. GRAB cannot exactly predict the degree of distant relationships but can in cases identify the existence of a relationship, and does very well in determining if a pair is unrelated.

### Comparison with existing approaches

**Figure 4:**
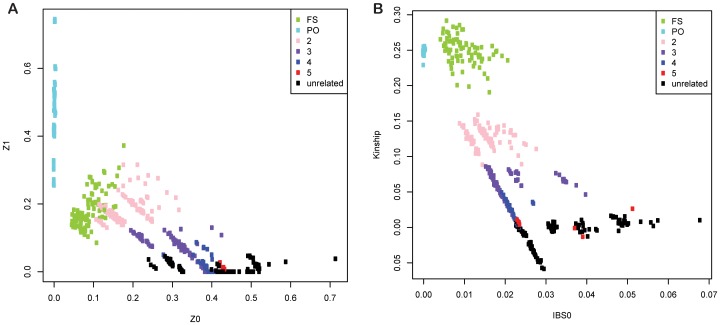
Performance of (A) PLINK and (B) KING on true families. Reported relationships are illustrated by different colors. PLINK's output Z0 P(IBD = 0) and Z1 P(IBD = 1) are plotted, KING's output IBS0 (proportion of zero IBS) and kinship coefficient are plotted. Relationship must be estimated by visualization of the clusters in the graph.

We compared GRAB with PLINK v1.07 [7], KING v1.1 [8], and ERSA 1.0 [10] for real whole-genome sequenced families. PLINK and KING provide genome-wide average measurements of IBS; Individual pairs with same relationship degree tend to cluster together (Figure 4). Linear discriminant analysis was used to classify relationship from PLINK/KING's output metrics, and leave-one-out cross-validation was used to estimate prediction accuracy (Table 3). Deriving accurate relationships from these estimation algorithms is sensitive to the variation around the expected mean proportion of genome-wide sharing, and therefore it can be challenging to classify more distant relationships. PLINK clearly identifies 1st-degree or 2nd-degree relationships, but only detects 52% 3rd-degree relations (Table 3). KING can correctly predict 86% 3rd-degree relationships, but cannot distinguish between 4th-degree and unrelated. In contrast, GRAB and ERSA directly outputs the computed relationships. GRAB correctly predicts 93% 3rd-degree, and less than 5% of 5th-degree relations are mispredicted as unrelated. ERSA 1.0 can predict 1st-degree through 9th-degree relationships, but its accuracy for full-sibling, 2nd-degree and unrelated individuals are lower than GRAB (Table 3). To resolve these problems and extend ERSA to WGS data, ERSA 2.0 is under development.

**Table 3:** Comparison of multiple relationship estimation methods on real WGS families.

Relationship degree	GRAB	PLINK	KING	ISCA+ERSA1.0	Phasing+GERMLINE+ERSA1.0
**Full sibling**	1 (1)	0.77 (1)	0.91	0.61 (1)	0.88 (1)
**Parent offspring**	1 (1)	0.78 (1)	1 (1)	0.99 (1)	1 (1)
**2nd-degree**	0.97 (1)	0.85 (0.98)	0.93 (1)	0.67 (1)	0.58 (1)
**3rd-degree**	0.93 (1)	0.52 (0.73)	0.86 (0.97)	0.86 (0.99)	0.87 (0.98)
**4th-degree**	0.97 (1)	0 (0.19)	0.37 (0.59)	0.83 (0.98)	0.84 (1)
**5th-degree**	0.57 (0.57)	0 (0)	0 (0.04)	0.56 (0.92)	0.48 (0.90)
**unrelated**	0.98	0.81	0.93	0.67	0.37

Values in the table are the percentage of correct predictions. Values in parentheses are the percentage of predictions within one degree of the true relationship.

We further compared the computational time of these methods (Table 4). GRAB on whole-genome sequenced individuals is about 1 minute for a single pair and 14 minutes for all 78 comparisons between 13 individuals. KING and PLINK are faster, can be finished within 4 minutes even for 78 individual pairs. GERMLINE+ERSA is slow if counting the phasing time against it. But if the genotype data have already been phased, it is quite quick and the running time does not obviously increase with same size. ISCA+ERSA gets similar accuracy with GERMLINE+ERSA and does not need phasing, while the time complexity of ISCA is the square of sample size.

**Table 4:** Computation time in minutes for GRAB and other methods on sets of whole-genome sequences, using standard input data formats.

Number of individuals	Number of individual pairs	GRAB	PLINK	KING	ISCA+ERSA1.0	Phasing+GERMLINE+ERSA1.0
**2**	**1**	1∶22	1∶12	0∶17	14∶14	82+3
**13**	**78**	13∶50	3∶14	0∶32	1110	82+3

Time spent on transforming data formats is ignored. In this example, we expended 82 minutes to phase 13 individuals by BEAGLE (excluding any markers with a no-call rate greater than 5%). More accurate phasing would require more individuals and longer time.

## Discussion

GRAB employs windowing to smooth signal across local blocks of the genome. Some of the value of this “low-pass filtering” can be achieved by manual curation of the variants employed for relationship detection. Indeed, years of effort have gone into curation of genotyping panels such as those marketed by Affymetrix and Illumina. The resulting panels of common single-nucleotide polymorphisms (SNPs) tend to have been selected for low error rates as typed by the panel's particular technology, and also for Hardy-Weinberg equilibrium as referenced to legacy population-specific allele frequencies. The inclusion of SNPs in subsequent generations of these panels has changed, in part based on unknown proprietary criteria. SNPs that contribute poorly to market metrics such as typability tend to be pruned. Because of the high quality of these processes, SNPs included in current generations of such panels tend to be in Hardy-Weinberg equilibria in European populations and accurately typable by oligo-based hybridization techniques. The most significant drawback to the filtering processes used to reject SNPs from genotyping panels is that they leave voids in regions of genomes. WGS fills these voids by typing rare SNVs and by typing SNPs not passing filtering criteria. However, this void filling currently comes at a price of increased noise on average for individual SNVs – not necessarily because particular SNPs are less accurately typed than the identical SNPs on a genotyping panel, but because the SNVs not included on genotyping panels are on average inherently harder to type. Tools such as GRAB are therefore needed to enable the full value of WGS to be used in relationship detection. A future direction for algorithm development will be empirical training on aggregated data from thousands of whole genomes spanning diverse populations.

GRAB is designed for analysis of WGS data and achieves prediction accuracy that is similar to current approaches. GRAB can directly read whole-genome variant information from standard formats without requiring additional file conversion utilities or user-guided selection of appropriate markers. The algorithm has been trained specifically for WGS data and evaluates all variants in the genome, taking advantage of information from both rare and common variants. Although GRAB is optimized for WGS, it is also applicable to high-density genotyping arrays. GRAB can be integrated into workflows such as Genome Management System (github.com/systemsbiology/GMS), which is capable of parsing pedigree file and comparing reported relationship with GRAB's prediction. As GRAB does not need phased data or population background statistics, it can be used for sparsely studied populations. Computation time is moderate, mostly spent reading data and computing IBS fractions. There are multiple ways to improve relationship estimation algorithms including phasing genotypes and using more realistic models of IBS distribution, but these may come with increased computation or a loss of specificity. We recommend GRAB for detecting 1st to 5th-degree relationships. For estimation of the degree of distant relationships, when phasing data is not available, block detection with ISCA and interpretation by ERSA works well [15]. If a representative population is available, population-based phasing with GERMLINE [14] and interpretation by ERSA works well. If computation time becomes limiting during analysis of a very large cohort, as may be the case in association studies requiring sequestration of closely related individuals, KING efficiently detects closer-than-3rd-degree relationships.
